# Propensity to seek healthcare in different healthcare systems: analysis of patient data in 34 countries

**DOI:** 10.1186/s12913-015-1119-2

**Published:** 2015-10-09

**Authors:** Tessa van Loenen, Michael J. van den Berg, Marjan J. Faber, Gert P. Westert

**Affiliations:** Radboud University Medical Center, Scientific Institute for Quality of Healthcare (IQ healthcare), PO Box 9101 (114), 6500 HB Nijmegen, The Netherlands; National Institute for Public Health and the Environment (RIVM), PO Box 1, 3720 BA Bilthoven, The Netherlands; Department of Social Medicine, Academic Medical Center, University of Amsterdam, PO Box 22700, 1100 DE Amsterdam, The Netherlands

**Keywords:** Propensity to seek care, Health care seeking behavior, Primary care, ACSCs

## Abstract

**Background:**

Some people have a lower threshold to seek care for certain symptoms than others. This study aims to investigate what factors are associated with patients’ propensity to seek care. In addition, this study explores whether patients’ propensity to seek care is associated with their actual health care utilization. We hypothesized that higher scores for propensity to seek care will lead to more general practitioners (GP) consultations, but to lower rates of avoidable hospitalization.

**Methods:**

Propensity to seek care and GP utilization were measured by the Patient Experience Questionnaire of the QUALICOPC study, a survey among 61,931 patients that recently visited GP services in 34 countries. Propensity to seek care was estimated by two questions: one question focusing on health care seeking behavior for serious symptoms and the other question focused minor complaints. Data on country level rates of avoidable hospitalization for CHF, COPD, asthma and diabetes were obtained from the OECD health care quality indicators project.

**Results:**

Beside patient characteristics, various organizational factors, such as better accessible and continuous primary care, and better experienced communication between patient and GPs was associated with a higher propensity to seek care for both severe and minor complaints. A higher propensity to seek care was associated with a slightly higher health care utilization in terms of GP visits, with no differences between the severity of the experienced symptoms (OR 1.08 for severe complaints and OR 1.05 for minor complaints). At country level, no association was found between propensity to seek care and rates of avoidable hospitalization for CHF, COPD, asthma and diabetes, possibly due to low statistical power at country level.

**Conclusions:**

The organization of primary care and patients’ perceived communication with their GP were found to be highly correlated with patients’ decision to seek health care for minor or severe complaints, suggesting that characteristics of healthcare systems directly influence patients’ care seeking behavior, potentially leading to overuse or underuse of health services. However, we also observed that patients’ propensity to seek care is only weakly associated with more GP use.

**Electronic supplementary material:**

The online version of this article (doi:10.1186/s12913-015-1119-2) contains supplementary material, which is available to authorized users.

## Background

Insight into drivers of health care seeking behavior and their effects on health care utilization may contribute to the design of more efficient and cost effective healthcare systems. Undoubtedly, people respond differently to symptoms and they vary in utilization of care. Some will have a higher tendency to seek care while others will tend to delay seeking help. On the one hand, a high propensity to seek care can result into early monitoring of diseases and deterioration might be prevented. On the other hand, a high propensity to seek care can also lead to unnecessary use or overuse of GP services, especially when there is a high propensity to seek care for non-urgent complaints.

The decision to seek care results from a mix of cultural, social, economic, geographical and organizational determinants [1, 2]. For example, women are assumed to consult the general practitioner (GP) more often than men [3–5]. A common explanation for this difference is that women have a higher propensity to seek care because they have a lower threshold to admit illness [6]. Research on the association between ethnicity and socio-economic factors and health care seeking behavior shows mixed results [7, 8]. Less attention has been given to the influence of healthcare organization and a country’s healthcare system in general. For instance, in countries with less accessible primary care systems, people might delay seeking care because of access barriers or make inappropriate use of hospital resources.

To study why people do or do not use care, Andersen created a widely used behavioral model of health care utilization [9, 10]. The model helps to structure determinants of health care utilization into three main categories: population characteristics, external environment and health care systems. The population characteristics can be predisposing, enabling or expressing needs. Predisposing factors, such as demographics, social structure or beliefs, influence people’s attitudes towards illness and care. Enabling factors, such as individual’s income, insurance status, or accessibility, reflect the availability of health care services. If health care services are not available or not within reach, health care seeking behavior might be affected. Lastly, there must be a certain need for care in order to seek for it. So, patients must perceive a need for care, they have to respond to this need and the patient’s environment must enable the search for care. Andersen’s model also acknowledges the role of societal determinants, such as political and economic factors, as well as health system factors, such as resources and organization, on health care utilization. Based on this model of health care utilization, the first aim of this study is to get more insight into the different factors underlying individuals’ propensity to seek care using a multi-country cross-sectional study.

The second aim of this study is to research how patients’ propensity to seek care relates to health care utilization. It is hypothesized that patients with a higher propensity to seek care tend to visit their GP more often. It is expected that countries with a population with a higher propensity to seek care, have lower rates of avoidable hospitalizations for chronic diseases. It has been argued that chronically ill patients who seek care at an earlier stage of their disease can be monitored better and that exacerbation of their disease can be reduced, which may result in lower risk of potentially avoidable hospital admission. The conditions for which a hospitalization can be potentially avoided are often referred to as ambulatory care sensitive conditions (ACSC) and include diabetes and asthma [11, 12]. This study investigates the association between the propensity to seek care and the number of GP visits on patient level and the association between a population’s propensity to seek care and rates of avoidable hospitalization at country level.

## Methods

### Data collection

Data were collected within the QUALICOPC study (Quality and Costs of Primary Care in Europe), in which surveys were held among GPs and patients in 31 European countries (EU 27–except for France-, and FYR Macedonia, Iceland, Norway, Switzerland and Turkey) and 3 non-European countries (Australia, Canada, New Zealand). In each country, we aimed to get a nationally representative sample of GPs (target: *N* = 220; Cyprus, Iceland, Luxembourg and Malta *N* = 80) to fill out the questionnaires. Random sampling was used to select practitioners in countries where national registers of practitioners were available. In countries with only regional registers, random samples were drawn from regions that represent the national setting. If only lists of facilities in a country existed a random selection of these lists was made. Per practice or health center, one GP was eligible for participation. Information on participation rates can be found elsewhere (Groenewegen PP, Gress S, Schafer WL.: General practitioners’ participation in a large, multi-country combined general practitioner–patient survey: recruitment procedures and participation rate (submitted)), [13]. Ethical approval was acquired in accordance with the legal requirements in each of the 34 countries. An overview of the concerned ethics committees are provided in Additional file 1. Written informed consents from participants were also acquired in accordance with the legal requirements in each of the countries. Both GP and patient surveys were filled out anonymously. In nearly all countries, trained fieldworkers visited the participating GP practices to collect patient data using paper surveys. Fieldworkers were instructed to consecutively invite patients of 18 years or older, who had had a face-to-face consultation with the GP, to complete the questionnaire until 10 patient surveys were collected. Nine out of ten patients completed the questionnaire about their experiences in the consultation that had just occurred. The tenth patient filled out the questionnaire about patient’s values in primary care. In addition, each trained fieldworker filled out a short questionnaire about the practice facility, like access to the practice for disabled. A unique practice identification number links GP responses to the responses of 10 of his or her patients and the fieldworker survey, allowing for multi-level analyses of the data. Data collection took place between October 2011 and December 2013. The GP questionnaire was filled in by 7,183 GPs and the patient experiences questionnaire by 61,931. Details about the sampling procedures and questionnaire development were published elsewhere [14, 15].

### Propensity to seek care measure

The propensity to seek care was estimated by two questions of the patient experience questionnaire (Table 1). One question focused on health care seeking behavior for serious symptoms while the other question focused on expected benefits from the visit to the GP for minor complaints. Both questions were derived from existing questionnaires [16, 17].

**Table 1:** Scales based on two questions

Propensity to seek care (severe complaints)
*How important would it be for you to see a doctor if you had…?*
* 1) Weight loss of more than 2 kilograms in a month when not dieting*
* 2) Shortness of breath with light exercise or light work*
* 3) Chest pain when exercising*
* 4) Loss of consciousness, fainting or passing out*
* 5) Headache for more than one day*
* 6) Abdominal pain for more than one day*
* 7) Severe worries for more than a month*
Answering categories: “extremely important, rather important, somewhat important, and not important”.
Propensity to seek care (minor complaints)
*Do you expect to benefit from a GP visit for…?*
* 1) Stomach problems*
* 2) Shoulder and neck pain*
* 3) Feeling nervous*
* 4) Diarrhea*
* 5) Sore throat*
* 6) Headache*
* 7) Feeling tired*
* 8) Flu*
* 9) Feeling nauseous*
Answering categories were: yes, no

Scale scores were created using the ecometrics approach in which multilevel analyses are used to construct a contextual variable at a higher-level unit based on several related individual variables. This approach takes into account the differences in the number of respondents on which the estimation is based, individual differences in response to certain items, and for dependency among the items that measure the latent variable [18]. Scales were created both on country level, excluding patient and practice variation, as well as on patient level. A higher score on the scales indicates a higher propensity to seek care. Scales were created using MLwiN and ranged from 0 to 10. On patient level, the reliability scores were good, with a score of 0.73 for the scale on severe complaints and 0.95 for the scale on propensity to seek care for minor complaints . The reliability score of the scales on country level were low with a score of 0.59 for the scale on severe complaints and 0.69 for the scale on propensity to seek care for minor complaints . Scales were weakly correlated on country level (r = 0.35; *p* <0.05) or patient level (r = 0.28; *p* <0.05).

### Predictors for propensity to seek care

Analyses of the association between factors derived from Andersens’ model and of propensity to seek care were performed using linear multilevel analyses with three levels (patient, GP practice and country). These analyses were done using STATA 13. Both scales are negatively skewed, however residuals and random effects are normally distributed hence transformation was not necessary. The potential predictors were included in the model step-by-step. The final model is presented in this article; the other models are presented in Additional file 2. Population characteristics were derived from the QUALICOPC study, while economic and health system factors originated from secondary databases.

Patients need for health care was determined by self-rated health, measured in four categories varying from very good to poor. The following predisposing patient characteristics were included in the analyses: age, gender, educational attainment and ethnicity. Patients’ ethnic background was determined by the place of birth of the patient and the mother. Patients were considered ‘native’ if both or only the mother was born in the country. If the patient was born in the country but the mother in a foreign one, the patient was considered a ‘second generation migrant’. When both the patient and mother were born in another country, the patient was considered a ‘first generation migrant’. The educational attainment of patients was categorized into ‘high’ (post-secondary or higher), ‘middle’ (upper secondary education) and ‘low’ (no education, (pre)primary or lower secondary education).

Income, urbanization, and scales on accessibility, continuity and experienced doctor patient communication were included as enabling factors. Household income was categorized as ‘below national average’ , ‘around average’ or ‘above average’. Urbanization was measured at practice level: ‘big (inner) city’ , ‘suburb or town’ , ‘urban–rural or rural’. The access-scale was composed of five items about patients’ experience with access barriers, i.e.: opening hours are too restricted, no home visit when needed, practice is too far, had to wait long to speak to someone when calling, and patients do not know how to get out-of-hours services. Longitudinal continuity of care was composed of the following items: patient has own doctor, doctor knows about patient’s medical background and doctor knows about patient’s living situation. The patient-doctor communication scale included the following aspects: doctor was polite, listened carefully, looked at me, asked questions about my health problem and I could understand what the doctor explained. A higher score on the scales indicates better access, better doctor-patient communication and more longitudinal continuity. Scales were created with the ecometric approach.

Countries GDP (PPP) per capita and type of health care system were included in the final model [19, 20]. Three types of health care system were identified: National Health Service (NHS), Social Security based system (SHI) or transitional. Transitional health care systems are situated in Eastern European where the system is in transition from a Soviet Union system to a social security-based system [19].

### Health care utilization

The association between the number of contacts with the GP in the past six months and propensity to seek care was evaluated using logistic multilevel analyses with adjustment for gender, age and presence of a chronic disease. The number of contacts with the GP was measured in the patient experience survey by 4 categories: “this consult was the first in the past 6 months” , “once before this visit” , “2 to 4 times before this visit” , “5 times or more before this visit”. For the analyses the measure was dichotomized into: ‘0–1 visit before this one’ and ‘at least two visits before this one’.

For the analyses on the association between propensity to seek care and avoidable hospitalization rates, a negative binominal model was used whereby incidence rate ratios (IRR) were calculated. Rates of avoidable hospitalization for four chronic conditions, asthma, COPD, congestive heart failure (CHF) and diabetes (uncontrolled diabetes, long-term complications and short-term complications), were obtained from the OECD health care quality indicators [21]. Data on avoidable hospitalization for England were obtained separately from NHS England; the data had been collected in accordance with the definition of the OECD health care quality indicator data collection of 2012–2013. All hospitalization rates were at country level and age-sex standardized per 100.000 population.

Researching the relationship between propensity to seek care and avoidable hospitalization on country level has some methodological considerations. Since data linkage on the individual level was not possible, this part of the analyses could only be carried out on aggregated (country) level. As a consequence, the number of cases is relatively small (18–24 countries) leading to a low statistical power. Because of this limitation, both significance levels of 0.05 and 0.10 are presented in the results. The results emerging from the analyses are to be regarded as indicative and more research might be needed to confirm the results.

Analyses were controlled for hospital bed supply and disease prevalence. Analyses on COPD hospital admission were also controlled for countries’ smoking prevalence. All control variables were derived from secondary databases [21–23]. Additional file 3 gives a description of the variables including descriptive statistics, countries per variable and sources of all included variables.

## Results

### Variation in propensity to seek care between countries

Of the 61,931 patients who participated in the survey, 96.8 % answered the questions about propensity to seek care. For severe complaints, having chest pain when exercising (87.1 %) or loss of consciousness (94.7 %), was most frequently agreed on that a doctor’s visit was necessary. Half of the patients indicated it was important to see a doctor because of unintended weight loss (50.4 %). Variation between countries was found largest for shortness of breath with light exercise; almost all of the Romanian participants (94.3 %) found it important to see a doctor for this symptom versus 27.3 % of the Slovakian participants. The scale created for severe complaints had a mean score of 7.6 with a range of 2.9 till 9.6.

Regarding minor complaints, patients expected to benefit most from visiting the GP for stomach problems (89 %) and least for nervous feelings (58 %). Expecting to benefit from GP visit for feeling nervous varies highly between countries. While 17 % of the Slovakian patients expect to benefit from visiting the GP, this was true for 85 % of the Macedonian patients. The mean score on the scale for propensity to seek care for minor complaints was 8.2, ranging between 0.4 and 9.9.

**Fig. 1:**
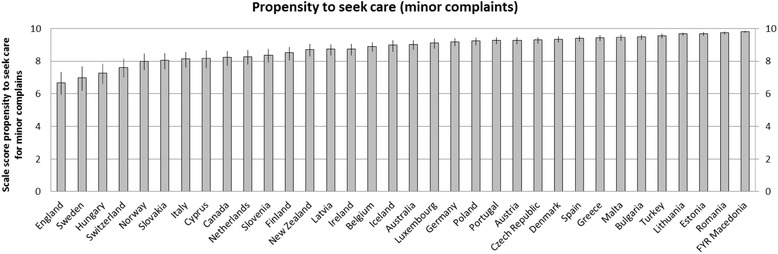
Variation in propensity to seek care for minor complaints between countries

**Fig. 2:**
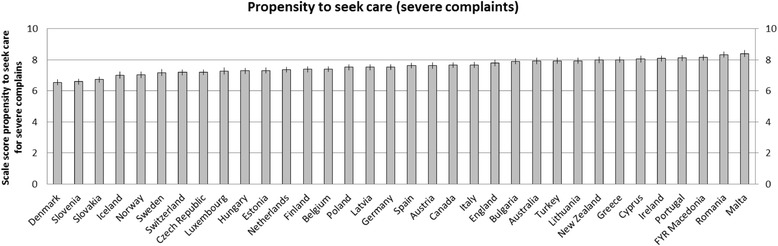
Variation in propensity to seek care for severe complaints between countries

Figures 1 and 2 present the variation between countries in propensity to seek care for severe and minor complaints respectively. The scale for propensity for minor complaints shows a wider variation than the scale for severe complaints. Interestingly, Denmark has the lowest score on severe complaints but one of the highest scores on minor complaints. For England, the opposite is the case. FYR Macedonia, Malta and Romania score high on both scales, while Sweden is scoring relatively low on both scales.

### Determinants for health care seeking behavior

Separate analyses were performed for severe complaints and minor complaints (Table 2). The final model of the multilevel analyses on severe complaints included 55,937 patients in 6784 GP practices in 34 countries. Adding new variables in the analyses hardly changed the effect sizes (see Additional file 1).

**Table 2:** Results of multilevel analyses to determine which factors are associated with propensity to seek care

Final models	Mean (SD) or %	‘Severe’ complaints			‘Minor’ complaints		
		(scale 0–10)			(scale 0–10)		
		B	LB	UB	B	LB	UB
*Intercept*		7.69**	7.35	8.03	8.33**	7.66	9.00
*Need factor*							
General health							
*-very good (ref)*	14.3 %						
*-good*	46.2 %	0.01	−0.01	0.04	0.10**	0.05	0.15
*-fair*	30.7 %	0.03	−0.001	0.06	0.15**	0.10	0.21
*-poor*	8.8 %	0.10**	0.06	0.14	0.19**	0.11	0.26
*Predisposing factors*							
Age/10^a^	51 (17.4)	0.06**	*0.05*	*0.07*	0.01	−0.004	0.02
Sex							
*- female*	61.1 %	0.05**	0.03	0.06	0.14**	0.11	0.17
Ethnicity							
*- native (ref)*	87.6 %						
*-second generation*	4.3 %	0.004	−0.04	0.05	−0.06	−0.14	0.02
*-first generation*	8.1 %	0.07**	0.03	0.10	0.04	−0.02	0.10
Educational attainment							
*-low (ref)*	28.0 %						
*-middle*	38.4 %	0.01	−0.02	0.03	−0.02	−0.07	0.02
*-high*	33.6 %	−0.03	−0.05	0.001	−0.09**	−0.14	0.04
*Enabling factors*							
Household income							
*Below average (ref)*	30.7 %						
*Around average*	57.0 %	0.02	−0.003	0.04	0.04	−0.002	0.07
*Above average*	12.3 %	0.03	−0.002	0.06	−0.06*	−0.12	0.001
Urbanization							
*Big city (ref)*	32,0 %						
*Suburbs, Town*	34.6 %	−0.005	−0.04	0.03	−0.01	−0.08	0.05
*Urban–rural, Rural*	33.4 %	−0.02	−0.05	0.02	0.03	−0.04	0.10
Access-scale (0–10)^a^	8.60 (0.28)	0.14**	0.11	0.17	0.35**	0.30	0.41
Communication-scale (0–10)^a^	9.52 (0.16)	0.11**	0.06	0.16	0.45**	0.35	0.55
Continuity-scale (0–10)^a^	9.33 (0.19)	0.37**	0.32	0.41	0.65**	0.56	0.73
*Health system*							
GDP (ppp) per capita/1000^a^	$33206,35 (12482,87)	−0.02**	−0.03	−0.01	−0.02	−0.05	0.003
Healthcare system							
*SHI (ref)*	23.5 %						
*NHS*	44.1 %	−0.03	−0.40	0.35	−0.47	−1.20	0.27
*Transitional*	32.4 %	−0.50*	−0.98	−0.01	−0.24	−1.19	0.71
*Variance Country (SE)*		0.161	(0.040)		0.617	(0.152)	
*Variance Practice (SE)*		0.221	(0.006)		0.822	(0.021)	
*Variance Patient (SE)*		0.956	(0.006)		3.199	(0.021)	
*Variance country (ICC)*			0.12			0.13	
*Variance practice (ICC)*			0.17			0.18	
*N (country)*			34			34	
*N ( practice)*			6784			6785	
*N (patient)*			55937			55417	

B = beta coefficient, LB = lower limit, UB = upper limit, ICC = intraclass correlation, ref = reference category

^*a*^ *Variables are centered around grand mean,* *P <0.05 **p <0.001

For severe complaints, a higher need for care in terms of poorer self-rated health was associated with higher propensity to seek care compared to those with lower need. Furthermore, several predisposing factors were significantly associated with higher propensity to seek care: female patients, older patients and first generation migrants tend to have higher scores on the propensity scale, although, the effects are small (0.05, 0.06 and 0.07 respectively on a scale of 0 to 10). Of the enabling factors, experiencing better access, better communication with the doctor and a higher continuity are strongly associated with having higher propensity to seek care. Especially patients experiencing high continuity with their primary care provider (having an ‘own’ doctor who knows something about their medical background and living situation), have a higher propensity to seek care (β 0.37, 95 % CI 0.32–0.41). Patients in countries with a social security based healthcare system tend to have a higher propensity to seek care for severe complaints.

For minor complaints, multilevel analyses showed that the poorer a patient’s health, the higher the propensity to seek care was. Yet, the magnitude of the effect was small with a β 0.19 (95 % CI 0.11–0.26) for patients with poor self-rated health compared to those with very good self-rated health. Female patients have higher propensity to seek care, although the effect is small (β 0.14; 95 % CI 0.11–0.17). Patients with a high educational attainment have a lower propensity to seek care compared to those with lower education. Other predisposing factors were not associated. The more accessible the GP, the higher the propensity to seek care for minor complaints. Patients who experience the communication with the doctor as good or experience a high degree of continuity also have higher propensity to seek care. Neither the type of healthcare system nor economic factors affected the patients’ propensity to seek care for minor complaints.

Remarkably, in both analyses of severe and minor complaints, even after adding several variables with significant effects, the explained variance remained more of less the same on all levels. For propensity to seek care for severe complaints, the intraclass correlation (ICC) of the final model is 0.17 at GP practice level and 0.12 on country level compared to an ICC of 0.16 at GP practice level and 0.15 on country level in the empty model. For minor complaints this is 0.18 on GP practice level and 0.13 on country level in the final model compared to an ICC of 0.17 at GP practice level and 0.15 on country level in the empty model.

### Health care seeking behavior and health care utilization

There was a significant but small positive association between self-reported GP utilization and the propensity to seek care for both severe and minor complaints. Patients with higher tendency to seek care for both severe (OR 1.08; 95 % CI 1.07–1.10) and minor complaints (OR, 1.05; 95 % CI 1.04–1.06) are more likely to have visited the GP at least twice in the past 6 months, when corrected for gender, age, and the presence of a chronic disease.

A higher propensity to seek care was associated with a reduced risk of hospitalization for uncontrolled diabetes (Table 3). In addition, a high propensity for minor complaints was associated with a reduced risk of hospitalization for asthma. Both results were significant statistically borderline.

**Table 3:** Results of negative binomial model avoidable hospitalization and minor complaints and severe complaints

	Severe complaints (scale 0–10)		Minor complaints *(scale 0–10)*	
	IRR	95 % CI	IRR	95 % CI
Asthma^a^ *(n = 19)*	0.72	0.37–1.42	0.70*	0.48–1.03
COPD^b^ *(n = 18)*	0.82	0.53–1.25	0.91	0.75–1.10
Diabetes: Long-term complications^a^ *(n = 23)*	1.34	0.84–2.15	1.07	0.82–1.40
Diabetes: Short-term complications^a^ *(n = 23)*	1.69**	1.15–2.49	1.11	0.87–1.43
Diabetes: Uncontrolled^a^ *(n = 21)*	0.53*	0.28–1.01	0.96	0.69–1.33
Congestive heart failure^c^ *(n = 24)*	1.12	0.77–1.65	0.99	0.81–1.20

IRR = incidence rate ratio

^a^adjusted for hospital bed supply, disease prevalence; ^b^adjusted for hospital bed supply, disease prevalence, smoking prevalence; ^c^adjusted for hospital bed supply

*p <0.10 **p <0.05

By contrast, a higher propensity to seek care for severe complaints was correlated with a higher risk for hospitalization for short-term complications of diabetes (IRR 1.69, *p* <0.05). Every increase on the scale in propensity to seek care for severe complaints is increasing the rate of avoidable hospitalization for short-term diabetes complications with a factor 1.69. It should be noted that in this analysis the country with the lowest score on the propensity scale (Denmark) and the country with the highest score (Portugal) are only 1.6 point departed from each other on a scale of 0 to 10.

## Discussion

In this study, we found that countries differ in their population propensity to seek care, although the variation is small. The tendency of patients to seek care for severe complaints is more equal across countries than patients’ propensity to seek care for minor complaints. Across all countries, patients acknowledge the importance of visiting a doctor for more severe complaints.

It has been argued that a patients’ health care seeking behavior is the result of cultural, social, economic, geographical and organizational determinants, indicating that not only the patients themselves but also the environment has an impact on patients’ health seeking choices [1]. The present study tried to explain the variation between patients’ propensity to seek care by using Andersen’s behavioral model of health care utilization that states that need, predisposing and enabling factors influence patients’ health care utilization. As expected, patients in more need, measured by self-rated health, have a higher propensity to seek care for both severe symptoms and minor symptoms. Furthermore, multiple predisposing factors such as age, gender, ethnicity and educational attainment are associated with a patient’s propensity to seek care. These results are in line with a previous study showing that patients aged 65 and over have a higher propensity to seek care [8]. However, other research showed that patients’ ethnicity, gender and socio-economic status were not related to health care seeking behavior in response to clinical vignettes [7]. A study showed that the association between for instance gender and health care seeking behavior depends on which symptom is researched [3, 24]. Our study also showed that the difference between men and women is smaller for severe complaints than for minor complaints. This is also the case for several other predisposing factors. Overall, it is clear that there are many patient characteristics contributing to patients’ decision to seek care.

Most interestingly, patients’ propensity to seek care is highly associated with their experience with the communication with GPs and the way primary care is organized. Patients experiencing better access, continuity and communication with the GP show a higher propensity to seek care. This was especially the case for propensity to seek care for minor complaints. Propensity to seek care for minor complaints was measured by asking patients whether they thought to benefit from visiting the GP for several non-urgent symptoms. Expecting to benefit from a GP depends highly on patients’ perception of the quality of their GP.

Patients within healthcare systems with accessible and continuous primary care are inclined to consult the GP earlier, which may lead to earlier detection and timely treatment of symptoms, and prevent deterioration of illness. However, no access barriers to the GP can easily lead to unnecessary and overuse of services. Finding the right balance is a challenge.

Looking at the issue of overuse of services, the question rises how patients’ health seeking behavior affects health care utilization. We hypothesized that higher scores for propensity to seek care will lead to more GP consultations, but to lower rates of avoidable hospitalization. The results showed that a higher propensity to seek care is indeed correlated with more GP consultations, but the effects are small. This was also found in other studies [8, 25]. Another study showed that individuals’ perceptions on inappropriate health services use are unlikely to have an effect on help-seeking behavior [26]. The decision to actually seek care when experiencing symptoms is influenced by more than only the propensity to seek care.

For most types of avoidable hospitalization, no association was found with countries’ propensity to seek care score. For admission rates for diabetes short-term complications, results contradicting to our hypotheses were found for country-level propensity to seek care score for severe complaints. The association implies a 69 % increase in risk for avoidable hospitalization with a higher country level propensity to seek care score. This indicates that a higher population propensity to seek care for serious complaints is associated with increased admission rates for short-term complications of diabetes, even when controlled for hospital bed supply or diabetes prevalence. Two previous studies [15, 27] on this topic also showed that propensity to seek care was not significantly associated with ACSCs, while another study [28] found that people in areas with high ACSC admission rates tended to delay outpatient care longer than in areas with low ACSC admission rates, which is contradicting to our findings. A literature review showed that strong primary care in terms of adequate physician supply and long-term relationships between patient and provider lowers the risk of avoidable hospitalization [29]. The current study, however, showed that increased accessibility and continuity are associated with higher propensity to seek care and that a higher country level propensity to seek care is not necessarily associated with lower rates of avoidable hospitalization for asthma, COPD, CHF or diabetes. Although these results should be approached with caution, they are in line with a previous study in California on a more detailed level [16].

### Strengths and limitations

A limitation of our study is that patients received a questionnaire after visiting the GP. By implication, patients with a low propensity to seek care are less likely to be included in the survey. This is also the case for people who experience access barriers or for people in good health. Ultimately, the results of our study are limited to people who actually use GP services. Still, a major benefit of this survey is that it results in a unique dataset about experiences of patients and GPs in 34 countries.

Another limitation is that the analyses of avoidable hospital admissions for ACSC were performed on country level and that not all countries were included. In our case, we would have liked to perform the analyses on patient level. However, avoidable hospitalizations were not measured on patient level because the prevalence of avoidable hospitalization is low, for example the mean prevalence of asthma in the included countries was 49 per 100.000. An individual level analysis would not be feasible at such international level with any dataset available. Therefore, we used an aggregated measure on a higher level. Consequences of performing analyses on country level are that the number of observations was small whereby there is low statistical power and that the reliability of the propensity to seek care scales on country level was low. Aggregating the data to such high level has the consequence that conclusions might be easily be biased by ecological fallacy. These methodological considerations do not allow us to draw firm conclusions about the relationship between propensity to seek care and avoidable hospitalization at individual level. Nevertheless, we think that propensity to seek care measured at individual level is a fair proxy for cultural differences in the tendency to seek care at country level. At country level we did not found the relationship between propensity to seek care and avoidable hospitalizations. Based on our data we cannot rule out the possibility that at individual level there is a relationship between propensity to seek care and avoidable hospitalization. To our knowledge this is the first explorative study attempting to research the association between propensity to seek care and rates of avoidable hospitalizations for several conditions in such large international context.

## Conclusions

This study shows that the propensity of patients to seek care when having symptoms varies across countries. Indeed, several patient characteristics correlate with the decision to seek care. Further, patients’ experiences with the organization of primary care are highly associated with care seeking behavior. The better patients experience accessibility, continuity of primary care and the communication with their GP, the higher their tendency to seek care for both severe and minor complaints. Hence, differences in healthcare systems have an effect on patients’ decisions to seek or not to seek care and can be of importance when dealing with underuse or overuse. A higher propensity to seek care leads to a slightly higher GP use, whilst no association was found for country level propensity to seek care and avoidable hospitalization for several chronic conditions.

## Additional files

Additional file 1:
**Overview of ethics committees within the QUALICOPC project.** (PDF 273 kb) Additional file 2:
**Step-by-step results of multilevel regression analyses.** (PDF 681 kb) Additional file 3:
**Description of variables included in negative binomial regression analyses.** (PDF 268 kb)

## Abbreviations

ACSC: Ambulatory care sensitive condition
COPD: Chronic obstructive pulmonary disease
GP: General practitioner
ICC: Intraclass correlation
IRR: Incidence rate ratio
NHS: National health service
OECD: The organization for economic co-operation and development
SHI: Social security based system
