# Supplementary material for: Propensity to seek healthcare in different healthcare systems: analysis of patient data in 34 countries
Source: BMC Health Serv Res. 2015 Oct 9;15:465. doi: 10.1186/s12913-015-1119-2 (PMC4600318; doi:10.1186/s12913-015-1119-2)
Supplement: Additional file 1: — Overview of ethics committees within the QUALICOPC project. (PDF 273 kb) [file 12913_2015_1119_MOESM1_ESM.pdf]

Additional file 1: Overview of ethics committees within the QUALICOPC project.

Country	Ethics committee
**Australia**	The Australian National University (ANU) Human Research Ethics CommitteeUniversity of Western Sydney Human Research Ethics Committee; The Royal Australian College of General Practitioners (RACGP) National Research and Evaluation Ethics Committee.
**Austria**	Ethics committee of the Medical University of Vienna
**Belgium**	University Hospital Ghent - Commission for Medical Ethics
**Bulgaria**	The coordinator sent an official letter to the Ministry of Health which gave consent and support for the survey. The coordinator confirmed that there is no statutory requirement for ethical approval for this study.
**Canada**	All procedures were approved by behavioural research ethics boards (BREBs) located at the institution where each provincial lead investigator was affiliated.
**Cyprus**	National Bioethical Committee of Cyprus
**Czech Republic**	General University Hospital linked to the First Faculty of Medicine, Charles University in Prague
**Denmark**	The coordinator confirmed that there is no statutory requirement for ethical approval for this study.
**Estonia**	The national coordinator consulted with the Ethics Review Committee on Human Research of the University of Tartu. It was confirmed that there is no statutory requirement for ethical approval for this study.
**Finland**	The ethical committee of Pirkanmaa Hospital District.
**Germany**	Ethics Commision of the "Landesärtzenkammer Hessen".
**Greece**	Bioethical committees of seventy hospital.
**Hungary**	National Ethical Committee
**Iceland**	The Icelandic Bioethics Committee. A national committee under the Ministry of Welfare.
**Ireland**	Irish College of General Practitioners Research Ethics Committee – National Committee.
**Italy**	At Local Health Authorities level. Approval was requested from LHA Ethical Committees.
**Latvia**	Latvian Physicians Association Board of Certification
**Lithuania**	Kauno Regionus Biomedicininu Tyrimu Etikos Komitetas
**Luxembourg**	National committee of Research Ethic (CNER) in Luxembourg
**FYR Macedonia**	Medical Faculty Skopje, R.Macedonia
**Malta**	University of Malta Research Ethics Committee
**Netherlands**	The ethics committee of VU Medisch Centrum confirmed via an official letter that the research is outside the scope of the WMO Act (Medical Research Involving Human Subjects Act).
**New Zealand**	Northern regional committee (Northern Y) for the nationally coordinated Health and Disability Ethics Committees (HDEC).
**Norway**	The coordinator confirmed that there is no statutory requirement for ethical approval for this study.
**Poland**	Bioethics approval of Jagiellonian University
**Portugal**	Ethical committee of Lisbon and Oporto regions.; the National Commission for Health Data Safety.
**Romania**	Scientific Committee of CPSS
**Spain**	Research Units of Primary Care of the Autonomous Community in the Basque Country. In all other Autonomous Communities the study was approved at the Healthcare Area level.
**Slovakia**	The national coordinator consulted with the Council of the Slovak Society of General Practice. It was confirmed that there is no statutory requirement for ethical approval for this study.
**Slovenia**	National medical ethics committee
**Sweden**	Regional Research Ethics Committte
**Switzerland**	Ethical Committee of the University of Lausanne
**Turkey**	Ethical committee of Kartal Research and Education Hospital in Istanbul
**United Kingdom**	University of Lincoln School of Health and Social Care Ethics Committee; National Research Ethics Service
