# Supplementary material for: Propensity to seek healthcare in different healthcare systems: analysis of patient data in 34 countries
Source: BMC Health Serv Res. 2015 Oct 9;15:465. doi: 10.1186/s12913-015-1119-2 (PMC4600318; doi:10.1186/s12913-015-1119-2)
Supplement: Additional file 2: — Step-by-step results of multilevel regression analyses. (PDF 681 kb) [file 12913_2015_1119_MOESM2_ESM.pdf]

Additional file 2: Step-by-step results of multilevel regression analyses

'Severe' complaints (scale 0-10)	***Model-0***			***Model-1***			***Model-2***		
	B	*LB*	*UB*	*B*	*LB*	*UB*	*B*	*LB*	*UB*
Intercept	7.56‡	7.40	7.72	7.46‡	7.30	7.62	7.49	7.32	7.65
Need factor									
General health									
-very good (ref)									
-good				0.07‡	0.05	0.10	0.02	-0.004	0.05
-fair				0.15‡	0.12	017	0.04‡	0.01	0.07
-poor				0.23‡	0.20	0.27	0.10‡	0.06	0.14
Predisposing factors									
Age/10*							0.07‡	0.06	0.07
Sex									
- female							0.05‡	0.03	0.07
Ethnicity									
- native(ref)									
-second generation							0.01	-0.04	0.05
-first generation							0.05‡	0.01	0.08
Educational attainment									
-low (ref)									
-middle							0.02	-0.004	0.04
-high							-0.01	-0.03	0.02
Enabling factors									
Household income									
Below average (ref)									
Around average									
Above average									
Urbanization									
Big city (ref)									
Suburbs, Town									
Urban-rural, Rural									
Access-scale (0-10)*									
Communication-scale (0-10)*									
Continuity-scale (0-10)*									
Health system									
GDP (ppp) per capita/1000*									
Health care system									
SHI (ref)									
NHS									
Transitional									
Variance Country (SE)	0.221	(0.054)		0.222	(0.054)		0.224	(0.054)	
Variance Practice (SE)	0.226	(0.006)		0.226	(0.006)		0.225	(0.006)	
Variance Patient (SE)	0.991	(0.006)		0.987	(0.006)		0.969	(0.006)	
ICC country level		0.15			0.15			0.16	
ICC practice level		0.16			0.16			0.16	
N (country)		34			34			34	
N(practice)		7006			7006			7005	
N (patient)		60853			60517			58729	

** Variables are centered around grand mean,* † P<0.05 ‡p<0.001

B=beta coefficient, LB= lower limit, UB=upper limit, ICC=intraclass correlation, ref=reference category

'Severe' complaints (scale 0-10)	***Model-3***			***Model-4 (final)***		
	B	*LB*	*UB*	*B*	*LB*	*UB*
Intercept	7.50‡	7.34	7.67	7.69‡	7.35	8.03
Need factor						
General health						
-very good (ref)						
-good	0.01	-0.01	0.04	0.01	-0.01	0.04
-fair	0.03	-0.001	0.06	0.03	-0.001	0.06
-poor	0.10‡	0.07	0.14	0.10‡	0.06	0.14
Predisposing factors						
Age/10*	*0.06‡*	*0.05*	*0.07*	*0.06‡*	*0.05*	*0.07*
Sex						
- female	0.05‡	0.03	0.06	0.05‡	0.03	0.06
Ethnicity						
- native(ref)						
-second generation	0.003	-0.04	0.05	0.004	-0.04	0.05
-first generation	0.07‡	0.03	0.10	0.07‡	0.03	0.10
Educational attainment						
-low (ref)						
-middle	0.01	-0.02	0.03	0.01	-0.02	0.03
-high	-0.03	-0.05	0.001	-0.03	-0.05	0.001
Enabling factors						
Household income						
Below average (ref)						
Around average	0.02	-0.003	0.03	0.02	-0.003	0.04
Above average	0.03	-0.002	0.06	0.03	-0.002	0.06
Urbanization						
Big city (ref)						
Suburbs, Town	-0.005	-0.04	0.03	-0.005	-0.04	0.03
Urban-rural, Rural	0.02	-0.05	0.02	-0.02	-0.05	0.02
Access-scale (0-10)*	0.14‡	0.11	0.17	0.14‡	0.11	0.17
Communication-scale (0-10)*	0.11‡	0.06	0.16	0.11‡	0.06	0.16
Continuity-scale (0-10)*	0.37‡	0.32	0.41	0.37‡	0.32	0.41
Health system						
GDP (ppp) per capita/1000*				-0.02‡	-0.03	-0.01
Health care system						
SHI (ref)						
NHS				-0.03	-0.40	0.35
Transitional				-0.50†	-0.98	-0.01
Variance Country (SE)	0.224	(0.055)		0.161	(0.040)	
Variance Practice (SE)	0.221	(0.006)		0.221	(0.006)	
Variance Patient (SE)	0.956	(0.006)		0.956	(0.006)	
Variance country (ICC)		0.16			0.12	
Variance practice (ICC)		0.16			0.17	
N (country)		34			34	
N(practice)		6784			6784	
N (patient)		55937			55937	

** Variables are centered around grand mean,* † P<0.05 ‡p<0.001

B=beta coefficient, LB= lower limit, UB=upper limit, ICC=intraclass correlation, ref=reference category

'Minor' complaints (scale 0-10)	Model-0			Model-1			Model-2		
	B	*LB*	*UB*	*B*	*LB*	*UB*	*B*	*LB*	*UB*
Intercept	8.19‡	7.90	8.48	8.02‡	7.73	8.31	7.99‡	7.69	8.28
Need factor									
General health									
-very good (ref)									
-good				0.15‡	0.11	0.20	0.13‡	0.08	0.17
-fair				0.24‡	0.19	0.29	0.18‡	0.13	0.23
-poor				0.26‡	0.19	0.32	0.19‡	0.12	0.26
Predisposing factors									
Age/10*							0.02‡	0.01	0.03
Sex									
- female							0.16‡	0.13	0.19
Ethnicity									
- native(ref)									
-second generation							-0.08†	-0.16	-0.01
-first generation							-0.01	-0.08	0.05
Educational attainment									
-low (ref)									
-middle							-0.003	-0.04	0.04
-high							-0.07‡	-0.11	-0.02
Enabling factors									
Household income									
Below average (ref)									
Around average									
Above average									
Urbanization									
Big city (ref)									
Suburbs, Town									
Urban-rural, Rural									
Access-scale (0-10)*									
Communication-scale (0-10)*									
Continuity-scale (0-10)*									
Health system									
GDP (ppp) per capita/1000*									
Health care system									
SHI (ref)									
NHS									
Transitional									
Variance Country (SE)	0.735	0.180		0.734	(0.180)		0.745	(0.183)	
Variance Practice (SE)	0.839	0.021		0.836	(0.021)		0.839	(0.021)	
Variance Patient (SE)	3.322	0.020		3.311	(0.020)		3.274	(0.020)	
Variance country (ICC)		0.15			0.15			0.15	
Variance practice (ICC)		0.17			0.17			0.17	
N (country)		34			34			34	
N(practice)		7005			7005			7005	
N (patient)		60236			59903			58156	

** Variables are centered around grand mean,* † P<0.05 ‡p<0.001

B=beta coefficient, LB= lower limit, UB=upper limit, ICC=intraclass correlation, ref=reference category

'Minor' complaints (scale 0-10)	***Model-3***			***Model-4 (final)***		
	B	*LB*	*UB*	*B*	*LB*	*UB*
Intercept	8.01‡	7.72	8.31	8.33‡	7.66	9.00
Need factor						
General health						
-very good (ref)						
-good	0.10‡	0.05	0.15	0.10‡	0.05	0.15
-fair	0.15‡	0.10	0.21	0.15‡	0.10	0.21
-poor	0.19‡	0.11	0.26	0.19‡	0.11	0.26
Predisposing factors						
Age/10*	0.01	-0.005	0.02	0.01	-0.004	0.02
Sex						
- female	0.14‡	0.11	0.17	0.14‡	0.11	0.17
Ethnicity						
- native(ref)						
-second generation	-0.06	-0.14	0.02	-0.06	-0.14	0.02
-first generation	0.04	-0.02	0.10	0.04	-0.02	0.10
Educational attainment						
-low (ref)						
-middle	-0.02	-0.07	0.02	-0.02	-0.07	0.02
-high	-0.09‡	-0.14	-0.04	-0.09‡	-0.14	0.04
Enabling factors						
Household income						
Below average (ref)						
Around average	0.04	-0.002	0.07	0.04	-0.002	0.07
Above average	-0.06†	-0.12	-0.001	-0.06†	-0.12	0.001
Urbanization						
Big city (ref)						
Suburbs, Town	-0.02	-0.08	0.05	-0.01	-0.08	0.05
Urban-rural, Rural	0.03	-0.04	0.10	0.03	-0.04	0.10
Access-scale (0-10)*	0.35‡	0.30	0.41	0.35‡	0.30	0.41
Communication-scale (0-10)*	0.45‡	0.35	0.55	0.45‡	0.35	0.55
Continuity-scale (0-10)*	0.65‡	0.56	0.73	0.65‡	0.56	0.73
Health system						
GDP (ppp) per capita/1000*				-0.02	-0.05	0.003
Health care system						
SHI (ref)						
NHS				-0.47	-1.20	0.27
Transitional				-0.24	-1.19	0.71
Variance Country (SE)		0.733	(0.180)	0.617	(0.152)	
Variance Practice (SE)		0.822	(0.021)	0.822	(0.021)	
Variance Patient (SE)		3.199	(0.021)	3.199	(0.021)	
Variance country (ICC)		0.15			0.13	
Variance practice (ICC)		0.17			0.18	
N (country)		34			34	
N(practice)		6785			6785	
N (patient)		55417			55417	

** Variables are centered around grand mean,* † P<0.05 ‡p<0.001

B=beta coefficient, LB= lower limit, UB=upper limit, ICC=intraclass correlation, ref=reference category
