# Supplementary material for: Propensity to seek healthcare in different healthcare systems: analysis of patient data in 34 countries
Source: BMC Health Serv Res. 2015 Oct 9;15:465. doi: 10.1186/s12913-015-1119-2 (PMC4600318; doi:10.1186/s12913-015-1119-2)
Supplement: Additional file 3: — Description of variables included in negative binomial regression analyses. (PDF 268 kb) [file 12913_2015_1119_MOESM3_ESM.pdf]

**Additional file 3: Description of variables included in negative binomial regression analyses**

| **Dependent variables** | | **N** | **Included countries** | **Year** | **Source** | **Mean (range)** | **SD** |
|---|---|---|---|---|---|---|---|
| Avoidable hospitalizations | Asthma admission rate per 100.000 population (age-sex stand.) | 25 | AUT, BEL, CZE, DNK, FIN, DEU, HUN, ISL, IRL, ITA, LVA, LUX, NLD, NOR, POL, PRT, SVK, SVN, ESP, SWE, CHE, ENG, AUS, CAN, NZL | 2009-2012 | OECD | 48.7 (11.4-150.9) | 34.1 |
| | COPD admission rate per 100.000 population (age-sex stand.) | 25 | AUT, BEL, CZE, DNK, FIN, DEU, HUN, ISL, IRL, ITA, LVA, LUX, NLD, NOR, POL, PRT, SVK, SVN, ESP, SWE, CHE, ENG, AUS, CAN, NZL | 2009-2012 | OECD | 209 (70.5-365.0) | 82.5 |
| | Diabetes: Short-term complication admission rate per 100.000 population (age-sex stand.) | 23 | AUT, BEL, CZE, DNK, FIN, DEU, HUN, ISL, IRL, ITA, LVA, NLD, NOR, POL, PRT, SVN, ESP, SWE, CHE, ENG, AUS, CAN, NZL | 2009-2012 | OECD | 18.3 (7.8-37.5) | 7.8 |
| | Diabetes: Long-term complication admission rate per 100.000 population (age-sex stand.) | 23 | AUT, BEL, CZE, DNK, FIN, DEU, HUN, ISL, IRL, ITA, LVA, NLD, NOR, POL, PRT, SVN, ESP, SWE, CHE, ENG, AUS, CAN, NZL | 2009-2012 | OECD | 87.6 (19.4-249.9) | 61.4 |
| | Uncontrolled diabetes admission rate per 100.000 population (age-sex stand.) | 21 | AUT, BEL, CZE, DNK, FIN, DEU, HUN, ISL, IRL, ITA, LVA, NOR, POL, PRT, ESP, SWE, CHE, ENG, AUS, CAN, NZL | 2009-2012 | OECD | 43.2 (7.2-180.7) | 40.5 |
| | Congestive heart failure admission rate per 100.000 population (age-sex stand.) | 24 | AUT, BEL, CZE, DNK, FIN, DEU, HUN, ISL, IRL, ITA, LVA, NLD, NOR, POL, PRT, SVK, SVN, ESP, SWE, CHE, ENG, AUS, CAN, NZL | 2009-2012 | OECD | 245.16 (56.5-595.4) | 122.7 |
| **Independent variables** | | | | | | | |
| Hospital beds | Number of hospital beds per 100.000 population | 25 | AUT, BEL, CZE, DNK, FIN, DEU, HUN, ISL, IRL, ITA, LVA, LUX, NLD, NOR, POL, PRT, SVK, SVN, ESP, SWE, CHE, ENG, AUS, CAN, NZL | 2009-2012 | OECD | 4.6 (2.5-8.3) | 1.7 |
| Asthma prevalence | Population aged 15+ with asthma (%) (age-sex stand.) | 19 | AUT, BEL, CZE, DNK, FIN, DEU, HUN, IRL, ITA, LVA, LUX, NLD, POL, PRT, SVK, SVN, ESP, SWE, ENG | 2007-2010 | Eurostat Statistics Database | 6.5 (2-11) | 3.2 |
| COPD prevalence | Population aged 15+ with COPD (%) (age-sex stand.) | 19 | AUT, BEL, CZE, DNK, FIN, DEU, HUN, IRL, ITA, LVA, LUX, NLD, POL, PRT, SVK, SVN, ESP, SWE, ENG | 2007-2009 | Eurostat Statistics Database | 20.8 (13.1-31.9) | 4.5 |
| Diabetes prevalence | Population aged 20–79 with diabetes (%) (age stand.) | 23 | AUT, BEL, CZE, DNK, FIN, DEU, HUN, ISL, IRL, ITA, LVA, NLD, NOR, POL, PRT, SVN, ESP, SWE, CHE, ENG, AUS, CAN, NZL | 2011 | IDF | 6.5 (3.2-14.9) | 2.3 |
| Smoking | population aged 15+ daily smokers (%) | 18 | AUT, BEL, CZE, DNK, FIN, DEU, HUN, IRL, ITA, LUX, NLD, POL, PRT, SVK, SVN, ESP, SWE, ENG | 2006-2012 | OECD | 6.49 (3.2-14.9) | 2.3 |
